# Sufficiency of Isolated Vascularised Fibular Free Flaps for Pediatric Intercalary Lower Limb Reconstruction

**DOI:** 10.2106/JBJS.OA.25.00267

**Published:** 2026-01-16

**Authors:** Laura Saenz, Karen W. Wong, Kristen M. Davidge, Sevan Hopyan

**Affiliations:** 1 Division of Orthopaedic Surgery, The Hospital for Sick Children, Toronto, Canada; 2 Current Address: Orthopedics and Traumatology, Clinica Pediátrica Kidoz, San José, Costa Rica; 3 Division of Plastic and Reconstructive Surgery, The Hospital for Sick Children, Toronto, Canada; 4 Division of Plastic, Reconstructive, & Aesthetic Surgery, Department of Surgery, University of Toronto, Toronto, Canada; 5 Division of Orthopaedic Surgery, University of Toronto, Toronto, Canada

## Abstract

**Background:**

For intercalary defects after resection of pediatric sarcoma, an isolated vascularized free fibular graft (VFFG) offers a living bone option with the potential for durability but is underutilized due to concerns about mechanical sufficiency. By contrast, structural allograft is initially strong but is associated with a high complication profile with or without a composite VFFG (Capanna). Our aim was to compare the complications associated with allograft/VFFG composites to isolated VFFG.

**Methods:**

This is a retrospective comparison of 32 pediatric patients with primary femoral or tibial sarcoma. Reconstructions were categorized into 2 groups: allografts + VFFG (Capanna) (Group 1, n = 9) and “single barrel” isolated VFFG (Group 2, n = 23). Descriptive and inferential statistical analyses were performed.

**Results:**

No significant differences between the 2 groups were observed in age, sex, diagnosis, length of reconstruction, or follow-up (56 months (SD 29) for Group 1 and 53 months (SD 24) for Group 2). Group 1 participants exhibited a higher number of complications including the need for unplanned surgery (mean 2.9) compared with those in Group 2 (mean 1.6, p &lt; 0.01). Disparities in bone healing were notable. At the final follow-up, 46% in Group 1 had achieved full consolidation compared with 96% in Group 2 (p = 0.002). Graft fractures and hardware failures were not different between the groups despite full weight-bearing among all subjects, suggesting mechanical equivalency within the follow-up period. The mean numbers of unplanned surgeries per participant were 4.2 ± SD 4.4 and 2.3 ± SD 2.5 for Groups 1 and 2, respectively (p = 0.149).

**Conclusions:**

Isolated single barrel VFFG reconstructions are structurally sound and had fewer complications and faster union than composite allograft/VFFG composites.

**Level of Evidence:**

Retrospective cohort study Level III. See Instructions for Authors for a complete description of levels of evidence.

## Introduction

Intercalary resections for sarcoma are advantageous because they spare native articular surfaces. The resulting bony defect can be restored by a number of methods including allograft^1^, composite allograft vascularized-free fibular graft (VFFG) (Capanna)^2-6^, isolated VFFG^7,8^, two-stage cement followed by morselized bone graft (Masquelet)^9^, bone transport^10,11^, extracorporeal devitalized or radiated autograft^12^, and endoprostheses^13-15^. For children and adolescents, smaller skeletons, narrower soft tissue envelopes, growth potential, physical aspirations, and longevity of survivors are important considerations^16-18^.

Structural allograft restores similar anatomy as the missing bone segment and provides early structural integrity that is useful for achieving full weight-bearing^19-21^. However, the absence of blood supply is a disadvantage that obviates bone healing from fracture or infection. Unlike vascularized grafts that remodel thoroughly, allografts or nonvascularized autografts remodel only by a few millimeters at their junctions with living bone by creeping substitution^22^. Although there are exceptions in which bulk allograft has been observed to remodel more thoroughly^23^, in most cases, such grafts either do not remodel or resorb partially and weaken. When fracture and infection risks are optimally mitigated, allograft reconstruction survival can reach 85% at a median follow-up of nearly 8 years^24^. Allografts often entail lifelong activity restrictions as a precaution and are associated with a relatively high incidence of major revisions^25,26^. Fractures often require hardware and/or graft revision, and infections may require allograft debridement and potentially multistage revision.

To mitigate the undesirable complications of allografts while still retaining their initial structural benefit, composite reconstructions of allograft with vascularized fibular autograft have become relatively common^19,27-31^. In principle, as the allograft partially resorbs and weakens, the vascularized fibula within the allograft medulla or alongside the allograft will hypertrophy because it is loaded progressively. Fractures of vascular fibulae can heal, and antibiotics can be delivered to the living bone. Therefore, bony continuity can be salvaged even if the allograft must be debrided. There is conflicting information available about whether the time to bony union is shorter with composite grafts compared with allografts alone^4,32^. Somewhat unexpectedly, the total number of complications and survival of intercalary reconstructions is similar for both groups^32,33^. We hypothesized that the allograft component of a composite graft disproportionately accounts for complications.

An alternative technique to provide living bone but without the potential challenges of a structural allograft is to use an isolated intercalary vascularized fibular autograft. Such grafts are predominantly used in the upper limb with promising results^34^. When used for the lower limb, a “double-barrel” has traditionally been preferred with the assumption that mechanical support would be improved. Although cutting a single harvested fibula and folding it on its periosteum and vascular pedicle to generate a two-bone construct is feasible, the practice obviously limits the length of intercalary reconstruction that can be bridged with a single fibula. Emerging evidence suggests that “single-barrel” VFFGs offer an alternative option for lower limb salvage, thereby allowing for double the potential length of a two-bone construct. Single barrel VFFGs hypertrophy over time and allow return to day to daily activities and sports^2,7,8,35^. However, their use for the lower limb remains uncommon due to uncertainties regarding their mechanical sufficiency for massive defects, complication profiles, and donor site morbidity.

Our primary objective is to present and compare the complications among pediatric patients undergoing lower limb reconstructions after primary bone sarcoma resection using single-barrel VFFG or a reconstruction employing allograft, either as an isolated or a composite graft. We hypothesize that single VFFG is mechanically sufficient for massive intercalary bone defects and is associated with fewer complications than reconstructions employing allografts.

## Methods

Details, including management of limb length discrepancy and rehabilitation in Appendix.

This retrospective cohort study includes 32 children or adolescents younger than 18 years who underwent intercalary reconstructions for primary bone sarcoma of the tibia or femur at a single institution from 2005 to 2021 with a minimum of 18 months of follow-up.

### Surgical Approaches

#### Structural Reconstruction

The skeletal gap was spanned using long plates with screws in native bone before the insertion of intercalary bone graft. Adjustments were performed to ensure appropriate length, coronal, sagittal, and rotational alignments that were evaluated in comparison with the contralateral lower limb.

**Fig. 1:**
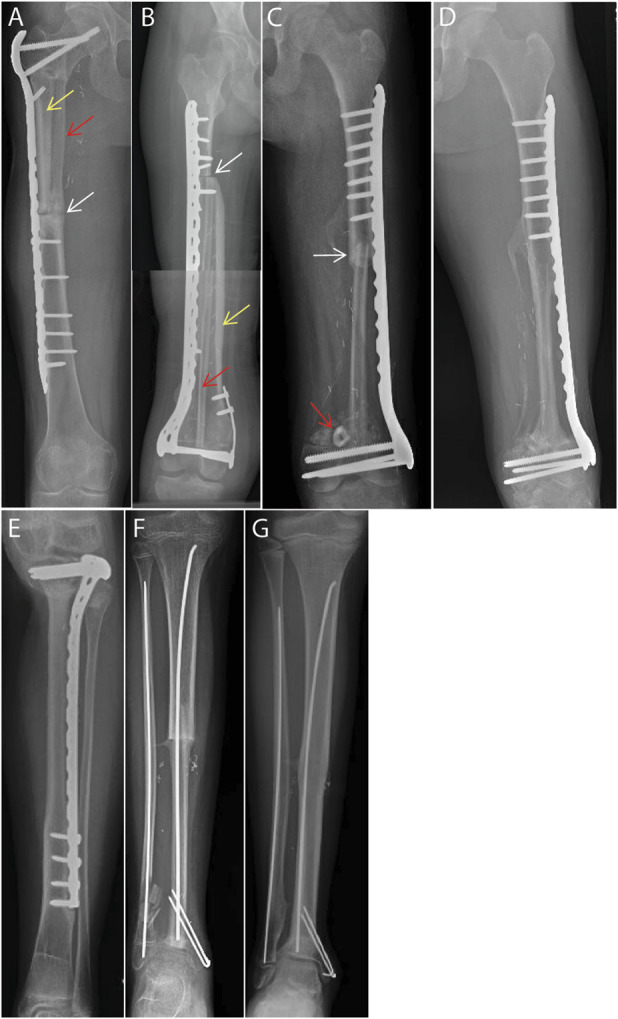
Representative examples of intercalary reconstructions. **Fig. 1-A** Proximal femoral composite graft with hemicortical allograft (yellow arrow) placed laterally and VFFG (red arrow) placed medially. Delayed union evident at 1 year (white arrow). **Fig. 1-B** Composite allograft (yellow arrow)/VFFG autograft (red arrow) exhibits a lack of bony consolidation at the proximal diaphyseal junction (white arrow) at 1 year. **Figs. 1-C and 1-D** Isolated distal femoral vascularized fibular autograft at 3 months that is intussuscepted proximally (white arrow) with excess graft length placed against the epiphysis (red arrow, **Fig. 1-C**). Same graft is fully consolidated at 1 year (**Fig. 1-D**). **Fig. 1-E** Isolated proximal tibial VFFG that is consolidated and hypertrophied at 3 years. **Figs. 1-**F **and 1-G** Isolated distal tibial VFFG with articular preservation at 3 months after cast removal with evidence of union and (**Fig. 1-F**) hypertrophied at 3 years postoperatively **Fig. 1-G**. VFFG = vascularized-free fibular graft.

Allografts and fibular autografts were intussuscepted and press-fit with native bone where possible. For composite grafts at the proximal femur, hemicortical allograft was placed lateral to the VFFG (Fig. 1-A). For composite grafts at the distal femur or proximal tibia, a longitudinal slot was cut into the posterior aspect of the structural allograft to widely accommodate the fibular autograft without compressing its periosteum or pedicle (Fig. 1-B). A small number of screws were placed into allografts for stability. No screws were placed into VFFGs to avoid injury to the vascular pedicle and to minimize the risk of a fracture. Single, lateral spanning plates were used for both types of reconstruction to minimize stress shielding and encourage hypertrophy. A supplementary flexible intramedullary was used for isolated VFFGs, and a supplementary short, malleable plate was used to compress distal femoral allografts to epiphyses (Figs. 1-C through 1-G).

#### Fibular Autograft Harvest and Microvascular Anastomosis

**Fig. 2:**
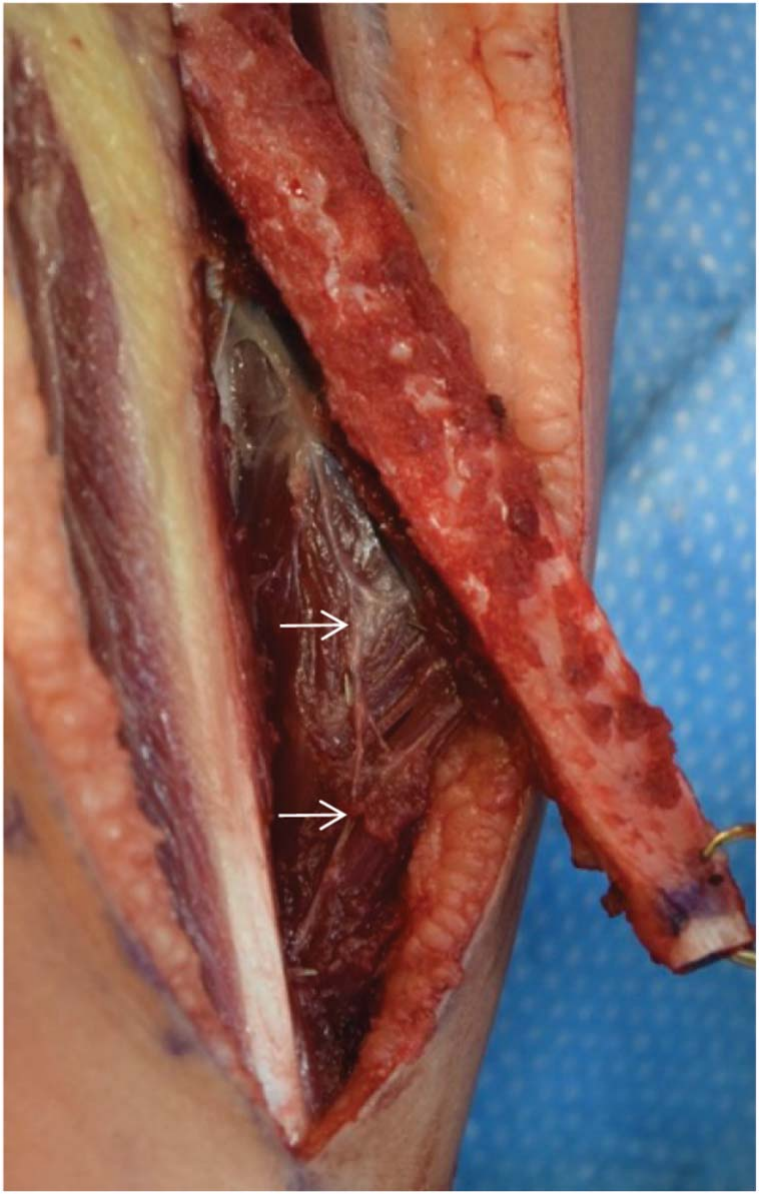
Vascularized fibular autograft being progressively separated from the FHL muscle origin. Note minimal muscle cuff left on the fibula, and preservation of the motor nerve branch or branches (white arrows) to the distal FHL that run alongside or with the pedicle. We postulate that these procedures minimize FHL contracture. FHL = flexor hallucis longus.

The fibular autograft was harvested from the unaffected contralateral limb simultaneously during tumor resection. Measures to minimize donor site morbidity included harvest of a minimal muscle cuff with the fibula; children have robust periosteum and this muscle cuff is not necessary to protect the periosteal sleeve. We also preserved nerve branches to the distal flexor hallucis muscle which often run alongside or within the pedicle. This serves to minimize the risk of muscle contracture (Fig. 2).

### Statistical Analyses

For normally distributed variables, the independent sample *t* test was applied, and for non-normally distributed variables, the Mann–Whitney *U* test was used to test for any significant differences in continuous variables.

## Results

### Baseline Characteristics

Intercalary reconstruction with a composite allograft/VFFG were designated as Group 1 (n = 9) and isolated VFFG as Group 2 (n = 23). There were no statistically significant differences between the 2 groups regarding age, sex, diagnosis (osteosarcoma vs. Ewing sarcoma), side affected, length of reconstruction (Group 1: mean ± SD, 19.7 ± 7.5 cm; Group 2: mean ± SD, 16.3 ± 4.0 cm), or follow-up from surgery to the last x-ray (Group 1: mean ± SD, 56 ± 29 months; Group 2: mean ± SD, 53 ± 24 months). Some of the shorter durations of follow-up were attributable to metastasis-related mortality. Chemotherapy for cases of osteosarcoma and Ewing sarcoma was consistent for each condition based on Children's Oncology Group protocols, and no participants received radiation. A higher proportion of patients in Group 1 had femoral sarcoma compared to those in Group 2 (p = 0.02) (Table I). This bias resulted from the stochastic presentation of sarcomas and by the team’s preference for composite reconstructions for the proximal femur.

**TABLE I:** Comparison of the Sociodemographic and Clinical Characteristics of Patients Between the Reconstruction Types

	Allograft-Fibula Composite		Isolated Vascularised Fibula		p
	Mean ± SD/Median (IQR)	N (%)	Mean ± SD/Median (IQR)	N (%)	
Age at surgery in years	10.5 ± 3.5/9 (8-13)		11.7 ± 3.2/13 (10-14)		0.34
Sex					
Female		6 (67)		15 (65)	0.94
Male		3 (33)		8 (35)	
Diagnosis					
Ewing Sarcoma		5 (56)		6 (26)	0.12
Osteosarcoma		4 (44)		17 (74)	
Bone affected					
Proximal femur		3 (33)		1 (4)	All femur vs. all tibia <0.01*†
Pan-diaphyseal femur		3 (33)		1 (4)	
Distal femur		2 (22)		8 (35)	
Proximal tibia		1 (11)		10 (44)	
Distal tibia		0		3 (13)	
Side					
Left		3 (33)		12 (52%)	0.35
Right		6 (67)		11 (48)	
Length of harvested autograft fibula (cm)	19.7 ± 7.5/18 (14.5-21.5)		16.3 ± 4.0/16 (13.3-18.0)		0.20
Months from surgery to last x ray (months)	56 ± 29/66 (28-73)		53 ± 24/49 (39-68)		0.84

SD = standard deviation and IQR = interquartile range.

* p < 0.05, significant.

† χ^2^ test.

### Complications

Complications such as soft tissue or wound necrosis, infection, hardware failure or pull out, painful hardware and graft fracture were relatively common in both groups. Except for bony union (discussed below), there were no significant differences in their incidence or the need for surgery to address individual types of complications between the 2 groups (Table II).

**TABLE II:** Comparison of Complications Between the Reconstruction Types

	Composite	Isolated	p
	Allograft-Fibula	Vascularised Fibula	
1. Wound necrosis with or without graft exposure (%)			
No	3 (33)	16 (70)	0.06
Yes	6 (67)	7 (30)	
2. Surgery for wound necrosis or graft exposure (%)			
No	3 (33)	16 (70)	0.06
Yes	6 (67)	7 (30)	
3. Infection (%)			
No	5 (56)	18 (78)	0.21
Yes	4 (44)	5 (22)	
4. Surgery for infection (%)			
No	5 (56)	19 (83)	0.12
Yes	4 (44%)	4 (17)	
5. Hardware failure, screw back out (%)			
No	6 (67)	15 (65)	0.94
Yes	3 (33)	8 (35)	
6. Surgery for hardware failure, screw back out (%)			
No	6 (67)	15 (65)	0.94
Yes	3 (33)	8 (35)	
7. Painful hardware requiring removal (%)			
No	7 (78)	20 (87)	0.54
Yes	2 (22)	3 (13)	
8. Graft fracture (%)			
No	6 (67)	18 (78)	0.51
Yes	3 (33)	5 (22)	
9. Surgery for graft fracture (%)			
No	7 (78)	20 (87)	0.54
Yes	2 (22)	3 (13)	
10. Partial fibular resorption (%)			
No	9 (100)	22 (96)	0.54
Yes	0 (0)	1 (4)	
11. Donor site problem (superficial dehiscence, infection, FHL contracture) (%)			
No	5 (56)	17 (74)	0.33
Yes	4 (44)	6 (26)	
12. Surgery for donor site problem (abscess, FHL contracture) (%)			
No	8 (89)	22 (96)	0.49
Yes	1 (11)	1 (4)	
Bony consolidation after 1 year postoperatively (%)			
None	2 (22)	1 (4)	
Partial	7 (78)	9 (39)	**< 0.001** * †
Full	0 (0)	13 (57)	
Full consolidation on last x-ray available (%)			
No	3 (33)	1 (4)	
Partial	2 (22)	0 (0)	**0.002** * †
Yes	4 (44)	22 (96)	
13. Bone grafting for nonunion or fibular resorption (%)			
No	6 (67)	22 (96)	**0.03** *
Yes	3 (33)	1 (4)	
14. Local recurrence (%)			
No	9 (100)	22 (96)	0.54
Yes	0 (0)	1 (4)	
15. Amputation (%)			
No	9 (100)	22 (96)	0.54
Yes	0 (0)	1 (4)	
Full weight-bearing at 1 year	7 (78)	22 (96)	0.13
Full weight-bearing at final F/U	9 (100)	23 (100)	
Clavien-Dindo-Sink classification			
II	0	8	
III	26	27	
IV	0	2	
No. of complications/participant	2.9	1.6	**< 0.01** * ‡
Total unplanned surgeries for complications per participant Mean ± SD/Median (IQR)	4.2 ± 4.4/3 (1-6)	2.3 ± 2.5/1 (1-3)	0.149

FHL = flexor hallucis longus, IQR = interquartile range, and SD = standard deviation.

Bold indicates statistically significant p values.

* p < 0.05 = significant.

† Fischer exact test.

‡ Mann–Whitney *U* Test.

Donor site complications were uncommon and primarily involved flexion contracture of the great toe. These contractures ceased after greater care was taken to preserve the motor branches coursing to the distal flexor hallucis longus (FHL) muscle belly during elevation of the fibular autograft. One contracture persisted despite stretching and required percutaneous FHL tenotomy for resolution. There were no cases of neurological deficit at the donor site such as numbness or weakness.

Significant differences were observed in bone healing. Although none of the patients in the allograft/composite Group 1 exhibited full consolidation (defined as all cortices on AP and lateral x-rays) at 1 year, 57% of those in the isolated VFFG Group 2 did so (p < 0.001). At the last follow-up, 44% of the reconstructions in Group 1 had achieved full consolidation in comparison with 96% in Group 2 (p = 0.002). One vascularized fibular autograft exhibited resorption of about half of its length and was managed with morselized autograft/allograft bone in a fashion analogous to Masquelet, leading to consolidation. More secondary bone grafting procedures were undertaken for nonunion in the allograft/composite Group 1 (3/9) versus the isolated VFFG Group 2 (1/23, p = 0.03).

A score combining all types of complications per participant was significantly higher for the composite Group 1 (2.9) compared with the isolated VFFG Group 2 (1.6, p < 0.01) (Table II). Complications were also classified according to the modified Clavien-Dindo-Sink Complication System. Most complications in both groups were Grade III, indicating that they were treatable but required surgery or unplanned hospital readmission. Eight cases in Group 2 were classified as Grade II which signified a deviation from the normal postoperative course and required outpatient treatment. Two Grade IV complications (limb-threatening) were observed in Group 2 that were due to the following (1) vascular spasm resulting in great toe necrosis and (2) local recurrence leading to through-knee amputation (Table II).

The number of unplanned operations for complications per participant was statistically not different but almost double for Group1 (mean ± SD: 4.2 ± 4.4, median (IQR [interquartile range]): 3 [1-6]) compared to the isolated VFFF Group 2 (mean ± SD: 2.3 ± 2.5, median [IQR]: 1 [1-3], p = 0.149) (Table II).

### Weight-bearing

At 1 year postoperatively, 7/9 (89%) patients in Group 1 and 22/23 (96%, p = 0.13) patients in Group 2 were fully weight-bearing. At the final follow-up, all patients in both groups were fully weight-bearing.

## Discussion

The merits of intercalary reconstruction can be considered from two perspectives—the value of articular preservation and the type of intercalary reconstruction material. Broadly speaking, articular preservation for metaphyseal or metadiaphyseal tumors is an alternative to intra-articular resection that would require different reconstructive options such as joint replacement or rotationplasty. The data in this study suggest that epiphyseal osteotomy is oncologically safe under the appropriate indications as has been demonstrated previously for physeal osteotomy^36^. Although we or others have not compared the merits of joint preserving vs. joint resecting approaches, pediatric endoprosthetic reconstruction is associated with high rates of deep infections, mechanical failures, and loosening that require major interventions or revisions^36-40^. The aspirational goal for articular preservation, especially in children, is that it will improve reconstruction longevity and diminish the need for major revisions over the long-term among survivors.

Based on the current comparison and on other published series of alternative nonliving intercalary materials, an isolated vascularized-free fibular graft is associated with a favorable complication profile^1-6,13-15,18,25,26^. Over 80% of allografts and composite reconstructions survive in the range of 10 to 20 years^24,32,33^. However, it remains unclear whether nonliving materials can be sustained over the 60- or 80-year timescale relevant to childhood sarcoma survivors.

Compared with allografts alone, composite grafts incorporating VFFGs have surprisingly not diminished the rate of complications requiring surgery such as nonunion, fracture, and infection^33^. One possibility to explain those findings is that the allograft portion of a composite graft contributes disproportionately to the incidence of complications. For example, infected allograft would still require debridement despite the presence of the VFFG, and intussuscepting the VFFG to optimize union may be challenging in the setting of a composite graft. It is also possible that presence of the allograft in composite grafts interferes with union of the internal vascularized fibula. In support of these concepts, elimination of the allograft in this series and by others^7^ diminished the rate of surgery for nonunions.

The number of debridements for graft exposure or infection and the overall rate of surgery for complications exhibit trends favorable to VFFG in this small series. Taken together, the lower complication score and the nearly two-fold difference in reoperation rate that favors isolated VFFG suggest that this study is statistically underpowered to effectively compare reoperation rates. Whereas surgery for graft exposure or fracture was not statistically different, interventions involving allograft are generally of greater acuity because they require debridement or revision. In our opinion, the reoperation information in this study is clinically significant partly due to the overall rate and partly to the generally lower acuity of the interventions for isolated VFFG problems. The need for additional surgery for isolated VFFG compares favorably with other intercalary reconstruction methods that restore living bone such as Masquelet and bone transport^41-45^.

The potential donor site morbidity of fibular harvest is rightly factored into considerations of VFFG use. However, problems, such as dorsal foot numbness, foot drop, and ankle instability, are largely preventable with precautions that are well recognized within the field^2,7,8,34,35^. The cases of FHL contracture in this series were encountered before a change in our practice. No such cases occurred after preservation of the motor nerve branch shown in Fig. 2, suggesting that denervation may account for the contracture, which is also preventable.

Comparative power and generalizability were limited because this study was conducted at a single site, incorporated small numbers of participants, and lacked race and ethnicity data. Variation of the fixation method based on the anatomical site was not profound as single spanning plates were used for both composite and isolated VFFG methods. Minor differences in fixation reflect inherent differences in the application of the two intercalary methods, and due to that, this study cannot precisely isolate the effect of the graft choice. The femoral site bias in the allograft group could conceivably have skewed the results favorably and unfavorably. For example, a relatively lower rate of complications may have been due to the greater muscle envelope of the thigh compared with the lower leg, and a higher rate of complications may have resulted from a relative mechanical challenge in stabilizing the femur compared with the tibia.

## Conclusions

An isolated single-barrel VFFG intercalary reconstruction is mechanically sufficient for full weight-bearing and the complication profile is superior to that of an allograft/VFFG composite. Whether the functional capacity of patients in high stress activities and sports is sufficient and the very long-term longevity is improved by this approach remain to be seen.

## Appendix

Supporting material provided by the authors is posted with the online version of this article as a data supplement at jbjs.org (http://links.lww.com/JBJSOA/B84). This content was not copyedited or verified by JBJS.
